# AIDS-defining illnesses among patients with HIV in Singapore, 1985 to 2001: results from the Singapore HIV Observational Cohort Study (SHOCS)

**DOI:** 10.1186/1471-2334-4-47

**Published:** 2004-11-12

**Authors:** Richard Bellamy, S Sangeetha, Nicholas I Paton

**Affiliations:** 1 Department of Infectious Diseases, Tan Tock Seng Hospital, 11 Jalan Tan Tock Seng, 308433, Singapore; 2 Current address: Nutrition and Public Health Intervention Research Unit, London School of Hygiene and Tropical Medicine, London, UK

## Abstract

**Background:**

The objective was to describe the causes of initial and overall AIDS-defining disease episodes among HIV patients in Singapore.

**Methods:**

A retrospective observational cohort study was performed of all adult patients seen at the national HIV referral center between 1985 and 2001. Data were extracted from the patients' records by ten trained healthcare workers. AIDS-defining conditions were established using predefined criteria.

**Results:**

Among 1504 patients, 834 had experienced one or more AIDS-defining diseases. The most frequent causes of the initial AIDS-defining episode were *Pneumocystis carinii *pneumonia (35.7%), *Mycobacterium tuberculosis *(22.7%) and herpes simplex (7.4%). In total 1742 AIDS-defining episodes occurred. The most frequent causes were *Pneumocystis carinii *pneumonia (25.1%), *Mycobacterium tuberculosis *(16.2%) and cytomegalovirus retinitis (9.5%).

**Conclusions:**

The most frequent causes of AIDS-defining illnesses in Singapore are similar to those reported in the West, prior to the introduction of anti-retroviral therapy. Opportunistic infections remain the most frequent AIDS-defining illnesses.

## Background

Cohort studies have provided valuable information on the clinical course of HIV infection in patients from Europe [1-16], North America [3,17-21], South America [22,23] and Africa [24-32]. The introduction of antiretroviral therapy has dramatically altered the incidence of AIDS-defining illnesses in the West. In the EuroSIDA study there have been substantial falls in the incidence and total number of AIDS-defining episodes between 1994 and 1998 [10]. During this period the proportions of reported AIDS-defining illnesses due to cytomegalovirus retinitis and *Mycobacterium avium *have decreased from 9% to 2% and 8% to 3% respectively [10]. The proportion due to non-Hodgkin's lymphoma has increased from 4% to 16% [10]. Substantial decreases in the incidence of disease caused by cytomegalovirus, *Pneumocystis carinii*, *Mycobacterium avium *and other opportunistic infections have also been observed in the United States [20,21].

Relatively few data are available on the course of HIV infection in Asian populations. In Bangkok between 1987 and 1993 the most frequent AIDS-defining diagnoses were extrapulmonary tuberculosis (22.8%), *Pneumocystis carinii *pneumonia (7.0%) and cryptococcal meningitis (10.9%) [33]. Little is known regarding whether the pattern of HIV-related conditions has changed following the introduction of antiretroviral therapy in Asia.

The Communicable Diseases Centre (CDC) in Singapore is the national reference centre for adult patients with HIV and AIDS and nationwide 94.9% of all Singaporean adult residents, who have ever been diagnosed, have been referred there. Underreporting of HIV does not occur as reporting occurs automatically from the reference laboratory. Records have been kept since the first case was identified in Singapore in 1985. Therefore the Singapore HIV Observational Cohort Study (SHOCS) contains almost the entire country's HIV experience. Nucleoside analogues have been available in Singapore since the early 1990s and protease inhibitors and non-nucleoside reverse transcriptase inhibitors since 1997. The usage of these drugs has been increasing annually and during 2000 and 2001 approximately 70–80% of patients regularly attending CDC were taking some form of anti-retroviral therapy.

## Methods

The details of the SHOCS cohort have been described previously [34]. The study was approved by the Ethics Committee of Tan Tock Seng Hospital. Data were extracted from the case notes of all Singaporean residents who were seen at CDC on or before 31^st ^December 2001, by ten trained healthcare workers. Probable and confirmed criteria were developed for the diagnosis of category C [34] and category B (AIDS-related complex) conditions, based on the 1993 guidelines of the United States' Centers for Disease Control and Prevention [35].

All disease episodes were initially determined by the data extractors and then checked by an infectious disease physician (RB). Diagnoses were not included if they did not fulfill the specified criteria even if there was a strong clinical suspicion of a particular condition. If there was insufficient evidence to satisfy the criteria for a specific diagnosis, a more general diagnosis was assigned, for example cerebral lesion (cause unknown) would be used if there was insufficient evidence to support a diagnosis of toxoplasmosis of the brain or primary cerebral lymphoma.

Median CD4 counts were calculated based on the sample taken nearest the time of diagnosis of the initial AIDS-defining condition. CD4 counts from 893 patients were used, as in 37 (4.0%) cases no suitable count was available. Data were entered into a computer database (Microsoft Access™) and checked for errors and inconsistencies. Statistical analyses were performed using Stata 7.0™. Each new or recurrent AIDS-defining condition counted as one event (i.e. a patient with three diagnoses would contribute three events). An AIDS-defining condition was counted as a separate additional event if it recurred six or more months after the initial diagnosis or in the case of tuberculosis, six or more months after treatment completion. To examine the effects of highly active anti-retroviral therapy (HAART) on the proportions of AIDS-defining illnesses due to each of the three most common infections (*Pneumocystis carinii *pneumonia, *Mycobacterium tuberculosis *and cytomegalovirus retinitis), comparisons were made between the pre-HAART era (1986 to 1995) and the established HAART era (2000 to 2001) using χ^2 ^tests.

## Results

Among the 1504 patients infected with HIV who were seen at CDC between 1985 and the end of 2001, 834 developed one or more AIDS-defining conditions. The most frequent initial AIDS-defining disease episodes were due to *Pneumocystis carinii *pneumonia (35.7% of total diagnoses), *M. tuberculosis *(22.7%), herpes simplex (chronic mucocutaneous) (7.4%) and candidiasis (esophageal or tracheobronchial) (6.9%) (table 1). The median CD4 count at which the initial AIDS-defining condition occurred was 27 (inter-quartile range 11 – 63). The median CD4 count was higher for *M. tuberculosis *infection (52, IQR = 18 – 110) and Kaposi's sarcoma (46.5, IQR = 23.5 – 227.5) than for *Pneumocystis carinii *pneumonia (16.5, IQR = 9 – 40), herpes simplex (25, IQR = 9 – 63) and extrapulmonary cryptococcosis (13, IQR = 6.5 – 39) (table 1).

1742 AIDS-defining disease episodes were recorded. The 10 most frequent AIDS-defining conditions were all of infectious etiology. There were 1658 first episodes of disease and 84 recurrences. The most frequent AIDS-defining diseases were *Pneumocystis carinii *pneumonia (25.1% of total diagnoses), *M. tuberculosis *(16.2%), cytomegalovirus retinitis (9.5%), candidiasis (7.8%), chronic mucocutaneous herpes simplex (8.2%) and disseminated *M. avium *(8.2%) (table 2). Several additional conditions could have included undiagnosed AIDS-defining diseases. The most common of these infections was pneumonia of unknown cause (117 cases), which is likely to include some patients with *Pneumocystis carinii *pneumonia (table 3). Weight loss of >10% body weight was a frequent problem and affected 383 (25.5%) patients (table 3).

Between 1996 and 2001 there was a continuous increase in the total number of AIDS-defining disease episodes occurring each year, due to the increase in the number of patients being followed up. There was no significant change in the overall proportion of AIDS-defining episodes caused by infections. *Pneumocystis carinii *and *M. tuberculosis *remained the two commonest causes. Between 1986 and 1995 they respectively accounted for 26.7% and 10.8% of all AIDS-defining disease episodes. During 2000 to 2001 the percentage of episodes caused by *Pneumocystis carinii *was not significantly different at 26.3% (Yates' χ^2 ^= 0.01, 2df, *P *= 0.94), but that due to *M. tuberculosis *had increased to 18.0% (Yates' χ^2 ^= 8.10, 2df, *P *= 0.004). There was no significant change in the percentage of episodes due to cytomegalovirus retinitis (9.7% between 1986 and 1995 and 8.5% during 2000 and 2001; Yates' χ^2 ^= 0.25, 2df, *P *= 0.62) (table 4).

## Discussion

### Comparison of HIV-associated morbidity with other populations

In the SHOCS cohort the proportion of conditions which were of infectious etiology remained very high throughout the study period. The distribution of conditions was similar to that seen in Western countries prior to the introduction of anti-retroviral therapy [1]. However the proportion of conditions caused by mycobacteria was higher than in Europe [1,12]. In Bangkok the most common AIDS-defining conditions were infections. Between 1986 and 1993 the most frequent AIDS-defining illnesses in Bangkok were extra-pulmonary tuberculosis (22.8%), cryptococcal meningitis (10.9%) and *Pneumocystis carinii *pneumonia (7%) [33]. Between 1993 and 1996 they were extrapulmonary cryptococcosis (38.4%), tuberculosis (37.4%), wasting syndrome (8.1%) and *Pneumocystis carinii *pneumonia (4.8%) [36]. *Pneumocystis carinii *pneumonia caused a much higher proportion of AIDS-defining diseases in Singapore than in Bangkok and cryptococcal meningitis occurred less frequently in Singapore.

### Potential sources of bias

The SHOCS cohort provides data on the experience of 94.9% of Singaporean residents who have been diagnosed with HIV since the epidemic began. There are good diagnostic facilities in Singapore and patients with HIV-related illness receive extensive investigations. Therefore Singapore offers an ideal location for collecting information on the causes of morbidity and mortality in persons with HIV in Asia. Medical record keeping is of a high standard in our hospital and this allowed accurate diagnoses to be assigned for AIDS-defining conditions which occurred throughout the AIDS era. To ensure consistency, data was extracted by carefully trained healthcare workers and diagnoses were assigned using predefined criteria. All AIDS-defining diagnoses and all causes of death were reviewed by the same infectious diseases physician (RB). Follow-up rates in our cohort are very high with only 5.8% of patients lost to follow-up at 12 months and 9.3% after 3 years. The SHOCS cohort therefore provides a unique source of information on HIV infection in an Asian country since the start of the epidemic.

In a retrospective study it is impossible to ensure that all patients received a full series of investigations in order to satisfy the study criteria for diagnosis of HIV-related conditions. As a consequence the criteria for making a probable diagnosis are unavoidably biased against certain conditions. For example if a patient undergoes a therapeutic trial of tuberculosis treatment he or she can be given the diagnosis of "probable *Mycobacterium tuberculosis*". However if a patient receives a successful trial of treatment for disseminated *M. avium*, the diagnosis is "probable mycobacteriosis (species unidentified)".

Differences in physicians' awareness of the link between HIV and opportunistic infections can influence diagnosis rates. For example, the increase in the proportion of AIDS-defining disease episodes caused by tuberculosis between 1986–1995 and 2000–2001 may have been due to greater awareness of the link between HIV and tuberculosis. Diagnosis rates for *M. avium *and *M. tuberculosis *may also have been affected by improvements in mycobacterial culture techniques.

## Conclusions

Despite the availability of anti-retroviral medication, opportunistic infections remain a common problem among HIV patients in Singapore. This may partly be explained by the high proportion of Singaporean patients, who present with advanced disease and very low CD4 counts. These patients often already have one or more AIDS-defining opportunistic infections at first presentation. Opportunistic infections may also occur before adequate immune-reconstitution can occur when HAART is commenced when the patient has a very low CD4 count. Studies in the West have shown that AIDS-defining illnesses occur more frequently among patients who have a CD4 count below 50 when anti-retroviral therapy is commenced than among those with higher CD4 counts [37,38]. Immune-reconstitution may also be impaired by difficulties in maintaining high levels of adherence, sub-optimal treatment regimens and unplanned treatment interruptions (due to adverse effects, financial or logistical reasons).

The current reductions in the cost of anti-retroviral therapy (Eg. due to generic manufacture) may enable high levels of anti-retroviral use to be achieved in many Asian countries. The experiences of the SHOCS cohort are likely to be repeated throughout Asia. Our results suggest that avoidable opportunistic infections may continue to occur even when high levels of anti-retroviral use are achieved. To reduce the burden of morbidity and mortality caused by these infections, efforts must be made to diagnose HIV infection earlier and to make the treatment of HIV and its associated opportunistic infections more affordable.

## List of abbreviations

SHOCS = Singapore HIV observational cohort study, CDC = Communicable Diseases Centre, AIDS = acquired immunodeficiency syndrome, HIV = human immunodeficiency virus, HAART = highly active anti-retroviral therapy.

## Competing interests

The authors declare that they have no competing interests.

## Authors' contributions

RB participated in the design of the study, supervised data extraction, performed the statistical analysis and wrote the manuscript. SS designed the database and supervised data extraction. NP participated in the design of the study and contributed to the statistical analysis and production of the manuscript. All authors read and approved the final manuscript.

## Pre-publication history

The pre-publication history for this paper can be accessed here:



## Figures and Tables

**Table 1:** The most frequent initial AIDS-defining conditions in 834 Singaporean patients.

Condition	Median CD4 (IQR^1^)	Confirmed	Probable	Total (%)
*Pneumocystis carinii *pneumonia	16.5 (9 – 40)	203	139	342 (35.7)
*Mycobacterium tuberculosis*	52 (18 – 110)	148	70	218 (22.7)
Herpes simplex, chronic mucocutaneous	25 (9 – 63)	26	45	71 (7.4)
Candidiasis, esophageal or tracheobronchial	21 (8.5 – 86)	25	41	66 (6.9)
Cytomegalovirus disease	25 (10 – 51)	28	12	40 (4.2)
Cryptococcosis, extrapulmonary	13 (6.5 – 39)	41	NA	41 (4.3)
Cytomegalovirus retinitis	15 (8 – 38.5)	0	37	37 (3.9)
*Mycobacterium avium*, disseminated	13 (5 – 26)	35	NA	35 (3.6)
Toxoplasmosis of the brain	29 (15 – 44)	1	31	32 (3.3)
*Mycobacterium*, species unidentified	31 (12 – 80)	0	16	16 (1.7)
Kaposi's sarcoma	46.5 (23.5 – 227.5)	8	4	12 (1.3)
Cryptosporidiosis, chronic intestinal	31 (4 – 56)	8	NA	8 (0.8)
Burkitt's lymphoma	34 (15.5 – 64)	8	NA	8 (0.8)
Wasting syndrome of HIV	36 (25 – 65)	NA	6	6 (0.6)
Progressive multifocal leukoencephalopathy	42.5 (24.5 – 56.5)	2	3	5 (0.5)
Histoplasmosis, disseminated	22 (4 – 138)	5	NA	5 (0.5)
Other^2^	-----	11	6	17 (1.8)
Total	27 (11 – 63)	549	410	959 (100)

^1^IQR = inter-quartile range. ^2^The category designated "other" includes conditions for which four cases or fewer were diagnosed. The number of initial AIDS-defining conditions is greater than the number of patients with AIDS because many patients presented with more than one condition. NA = not applicable.

**Table 2:** The most frequent conditions among 1742 overall category C episodes

Condition	Confirmed	Probable	New	Recurrent	Total (%)
*Pneumocystis carinii *pneumonia	251	196	399	28	437 (25.1%)
*Mycobacterium tuberculosis*	194	89	263	20	283 (16.2%)
Cytomegalovirus retinitis	0	166	166	NA^1^	166 (9.5%)
Candidiasis, esophageal or tracheobronchial	40	96	129	7	136 (7.8%)
Herpes simplex, chronic mucocutaneous	59	84	133	10	143 (8.2%)
*Mycobacterium avium*, disseminated	141	NA	138	3	141 (8.2%)
Cytomegalovirus disease	79	35	112	2	114 (6.5%)
Cryptococcosis, extrapulmonary	84	NA	78	6	84 (4.8%)
Toxoplasmosis of the brain	2	61	57	6	63 (3.6%)
*Mycobacterium*, species unidentified	1	33	34	0	34 (2.0%)
Kaposi's sarcoma	17	6	23	0	23 (1.3%)
Cryptosporidiosis, chronic intestinal	19	NA	18	1	19 (1.1%)
Lymphoma, primary cerebral	10	10	20	0	20 (1.1%)
Wasting syndrome of HIV	NA	16	16	0	16 (0.9%)
Encephalopathy, HIV-related	NA	10	10	0	10 (0.6%)
Pneumonia, recurrent bacterial	9	NA	8	1	9 (0.5%)
Burkitt's lymphoma	9	NA	9	0	9 (0.5%)
Progressive multifocal leukoencephalopathy	2	5	7	0	7 (0.4%)
Histoplasmosis, disseminated	6	NA	6	0	6 (0.3%)
*Mycobacterium scrofulaceum*, disseminated	5	NA	5	0	5 (0.3%)
Other^2^	16	1	17	0	17 (1.0%)

^1^Following an initial diagnosis of cytomegalovirus retinitis, recurrences were not recorded as additional episodes of category C disease. ^2^The category designated "other" includes conditions for which four cases or fewer were diagnosed.

**Table 3:** Conditions which may have been undiagnosed category C illnesses and important non-category C conditions

**Possible category C conditions**	**New**	**Recurrent**	**Total**
Pneumonia, cause unknown^1^	114	3	117
Non-Hodgkin's lymphoma, extracerebral^2^	25	0	25
Meningitis, cause unknown^3^	21	0	21
Dementia^4^	20	0	20
Cerebral lesion, cause unknown^5^	18	0	18
**Non-category C conditions**			
Weight loss, >10% bodyweight	379	4^6^	383
Pneumonia, bacterial	102	4^7^	106
*Salmonella *septicemia	86	0^8^	86
*Penicillium marneffei*, disseminated	5	1	6

^1^Some of these patients died before a diagnosis could be established and others recovered following a mixture of antibacterial and *Pneumocystis *treatments. Therefore the majority of these cases are likely to be due to bacteria or *Pneumocystis carinii *infections. ^2^Histology in these cases was not sufficiently specific to confirm a diagnosis of Burkitt's or immunoblastic lyphoma. ^3^These patients represent a diverse mixture of cases without a confirmed diagnosis, but they are believed to include mycobacterial, bacterial, viral and fungal infections. ^4^This excludes cases which fulfilled the diagnosis of HIV-related encephalopathy. ^5^Many of these cases died soon after diagnosis and are likely to include a large number of patients with toxoplasmosis of the brain. ^6^Recurrent weight loss indicates that weight was regained and subsequently lost again and not that 20% of body weight was lost. ^7^These cases of recurrence of bacterial pneumonia were not category C conditions as they occurred more than 12 months after the preceding case. ^8^Four cases of recurrent *Salmonella *septicaemia are included in the "other" category of table 3.

**Table 4:** Number of episodes per year for the 12 most frequent overall AIDS-defining conditions

Condition	1986–1995	1996–1999	2000–2001	Total
*Pneumocystis carinii *pneumonia	94 (26.7)	197 (23.6)	146 (26.3)	437 (25.1)
*Mycobacterium tuberculosis*	38 (10.8)	145 (17.4)	100 (18.0)	283 (16.2)
Cytomegalovirus retinitis	34 (9.7)	85 (10.2)	47 (8.5)	166 (9.5)
Candidiasis, esophageal or tracheobronchial	16 (4.5)	70 (8.4)	50 (9.0)	136 (7.8)
Herpes simplex, chronic mucocutaneous	27 (7.7)	71 (8.5)	45 (8.1)	143 (8.2)
*Mycobacterium avium*, disseminated	22 (6.3)	86 (10.3)	33 (5.9)	141 (8.1)
Cytomegalovirus disease	29 (8.2)	46 (5.5)	39 (7.0)	114 (6.5)
Cryptococcosis, extrapulmonary	19 (5.4)	34 (4.1)	31 (5.6)	84 (4.8)
Toxoplasmosis of the brain	19 (5.4)	24 (2.9)	20 (3.6)	63 (3.6)
*Mycobacterium*, species unidentified	7 (2.0)	14 (1.7)	13 (2.3)	34 (2.0)
Kaposi's sarcoma	15 (4.3)	6 (0.7)	2 (0.4)	23 (1.3)
Cryptosporidiosis, chronic intestinal	3 (0.9)	9 (1.1)	7 (1.3)	19 (1.1)
Total^1^	352 (100)	834 (100)	556 (100)	1742 (100)

This table shows the new and recurrent episodes combined. Figure in brackets are the percentages for each time period. ^1^Total also includes AIDS-defining conditions other than the 12 most frequent.
